# Early Transcriptomic Signatures of Immune Response Modulation Following Antiretroviral Therapy in HIV-Infected Patients

**DOI:** 10.3390/ijms262110678

**Published:** 2025-11-02

**Authors:** Ekaterina A. Stolbova, Anastasia V. Pokrovskaya, Andrey B. Shemshura, Dmitry E. Kireev, Alexey A. Lagunin, Boris N. Sobolev, Sergey M. Ivanov, Olga A. Tarasova

**Affiliations:** 1 Laboratory of Big Data Analysis for Digital Pharmacology, Department of Bioinformatics, Institute of Biomedical Chemistry, Pogodinskaya Street 10-8, 119121 Moscow, Russia; ekaterina.a.stolbova@yandex.ru (E.A.S.); smivanov7@gmail.com (S.M.I.); 2 Federal Budget Institution of Science “Central Research Institute for Epidemiology” of the Federal Service for Surveillance on Consumer Rights Protection and Human Wellbeing, Novogireevskaya Street, 3A, 111123 Moscow, Russia; pokrovskaya_av@mail.ru (A.V.P.); dmitkireev@yandex.ru (D.E.K.); 3 Department of Infectious Diseases with Courses of Epidemiology and Physiology, Medical Institute, Peoples’ Friendship University of Russia, 6 Miklukho-Maklaya Street, 117198 Moscow, Russia; 4 Federal Budget Public Health Institution “Clinical Center of HIV/AIDS Treatment and Prevention” of the Ministry of Health of Krasnodar Region, 204/2, Mitrofana Sedina Street, 350000 Krasnodar, Russia; shemsh@mail.ru; 5 Department of Bioinformatics, Pirogov Russian National Research Medical University, Ostrovityanova Street 1, 117513 Moscow, Russia; alexey.lagunin@ibmc.msk.ru; 6 Laboratory of Structure-Function Based Drug Design, Department of Bioinformatics, Institute of Biomedical Chemistry, Pogodinskaya St. 10-8, 119121 Moscow, Russia; boris.sobolev.05.52@mail.ru

**Keywords:** antiretroviral therapy, HIV, RNA seq, paired analysis, gene expression

## Abstract

Human immunodeficiency virus (HIV) remains a global public health challenge. Antiretroviral therapy (ART) improves outcomes by suppressing viral replication and enabling immune recovery, yet the early molecular mechanisms of immune-related transcriptional change after ART remain insufficiently characterized. We enrolled eight ART-naïve male patients with HIV aged 18–35. Peripheral blood mononuclear cells (PBMCs) were collected before and after 24 weeks of combination ART (TDF, 3TC, DTG) and underwent bulk RNA-seq (Illumina HiSeq 1500, Illumina, Inc., San Diego, CA, USA). Differential expression was assessed with DESeq2 (paired design); gene set enrichment analysis (GSEA), principal component analysis (PCA), hierarchical clustering, and protein–protein interaction (PPI) networks (STRING/NetworkX) explored functional patterns and transcriptomic shifts. We identified 87 differentially expressed genes, including 67 downregulated interferon-stimulated genes (e.g., IFI44L, ISG15, STAT1) and 20 upregulated transcripts, mostly pseudogenes related to ribosomal proteins. Functional enrichment revealed suppression of type I interferon and other antiviral signaling pathways. PCA and hierarchical clustering indicated a post-ART transcriptional shift. These findings suggest that early immune recovery following ART involves downregulation of chronic interferon-driven activation. This observation may correspond to partial restoration of T-cell functional capacity, reduced immune exhaustion, and a rebalanced antiviral immune environment.

## 1. Introduction

Human immunodeficiency virus (HIV) continues to pose a major global public health challenge. According to recent estimates by the World Health Organization, approximately 40.8 million individuals were living with HIV worldwide in 2024, with more than 1.3 million new infections reported annually [1]. HIV pathogenesis is characterized by progressive depletion and functional impairment of CD4^+^ T lymphocytes, leading to profound immune suppression, chronic immune activation, and persistent systemic inflammation [2].

The introduction of combination antiretroviral therapy (ART) has markedly improved clinical outcomes by effectively suppressing viral replication and partially restoring immune function [3,4]. The transcriptional mechanisms underlying these changes can help reveal rare and unknown mechanisms of CD4+/CD8+ response to treatment. A better characterization of ART-induced molecular reprogramming is essential for optimizing long-term immune recovery.

Transcriptomic profiling provides a powerful approach for investigating host immune responses at the molecular level [5,6]. Previous studies using microarray-based platforms have reported broad transcriptional alterations associated with HIV infection and ART, including genes involved in immune signaling, inflammation, apoptosis, and metabolic regulation [7,8,9,10].

RNA sequencing (RNA-seq) has emerged as a sensitive and comprehensive tool for transcriptome analysis, offering the ability to detect RNAs with greater resolution [11]. Despite its advantages, relatively few studies leveraged RNA-seq to investigate ART-induced transcriptional changes [12,13].

In the present study, we conducted a paired transcriptomic analysis of peripheral blood mononuclear cells (PBMCs) from ART-naïve male individuals in the early stage of HIV-infection, all receiving the same treatment regimen. This design enabled within-subject analysis of early immune responses to antiretroviral therapy. Paired analysis enables direct comparison of pre- and post-treatment samples from the same individual, thereby increasing the sensitivity for detecting differentially expressed genes [14]. By focusing on within-patient comparisons, we aimed to identify differentially expressed genes and elucidate key biological pathways with early immunological shifts induced by ART. Our findings contribute to a deep understanding of the transcription dynamics underlying ART-induced immune modulation and may inform strategies to optimize immune recovery in people living with HIV.

## 2. Results

### 2.1. Identification of Differentially Expressed Genes

Differential gene expression was assessed in PBMCs from eight patients before and after ART initiation.

All patients achieved full virological suppression (HIV-1 RNA < 50 copies/mL) after 24 weeks of ART, consistent with effective antiretroviral treatment. Time to viral suppression after ART initiation varies across populations, with some studies reporting suppression in over 80% of patients by 24 weeks [15,16]. The dynamics of CD4+ and CD8+ T cell counts differed across individuals. While most patients showed moderate increases in CD4+ T cell counts, two patients (P07 and P08) exhibited a decline, indicating a suboptimal immunological response. In particular, patient P08 demonstrated a significant inversion of the CD4/CD8 ratio due to pronounced CD8+ T cell expansion, suggestive of persistent immune activation. This observation is consistent with earlier studies indicating that CD4/CD8 ratio normalization may lag behind viral suppression and remains impaired in individuals with persistent immune activation or suboptimal recovery [17,18]. Given the small, clinically homogeneous cohort and the absence of confounding conditions, all patients were initially retained for analysis. However, to account for potential outlier effects, an additional analysis was performed with the exclusion of these two participants (P07 and P08).

**Figure 1:**
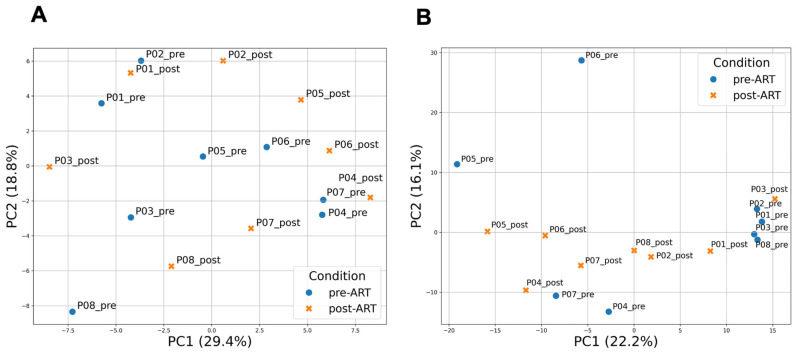
Principal component analysis (PCA) of gene expression profiles in PBMCs before and after ART. (**A**) PCA based on the 250 most variable genes. PC1 and PC2 explain 29.4% and 18.8% of the total variance, respectively; (**B**) PCA including all expressed genes passing the CPM filter. PC1 and PC2 explain 22.2% and 16.1% of the total variance, respectively. In both analyses, PCA was performed on log2(CPM + 1)-transformed expression values after filtering low-abundance genes (CPM ≥ 1 in ≥3 samples) and applying gene-wise z-scoring. Each dot represents a sample collected before (pre-ART) or after 24 weeks of ART (post-ART). Colors indicate ART status: blue for pre-ART and orange for post-ART samples.

Principal component analysis (PCA) was performed on the 250 most variable genes, selected after filtering out low-abundance features (CPM ≥ 1 in ≥3 samples) and log2 transformation. When retaining the top 250 most variable genes, the first two principal components (PC1 and PC2) explained 29.4% and 18.8% of the total variance, respectively, and revealed a partial separation of samples according to ART status (Figure 1A). PCA including all expressed genes explained a smaller proportion of variance (22.2% and 16.1% for PC1 and PC2, respectively) but produced clearer clustering of pre- and post-ART samples (Figure 1B). The third component (PC3) accounted for an additional 12.0% of the variance, and the PC1 versus PC3 projection is shown in Supplementary Figure S1.

**Figure 2:**
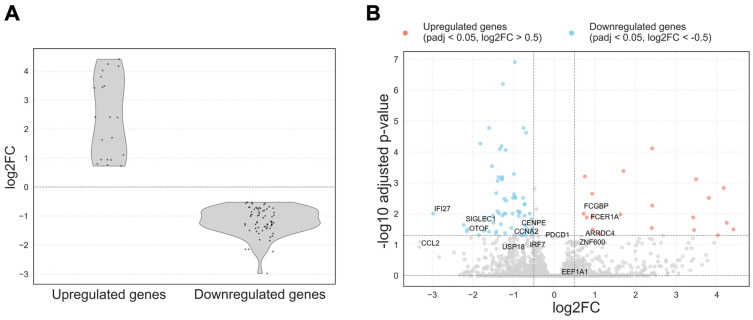
(**A**) Distribution of log2FC for differentially expressed genes with adjusted *p*-value < 0.05. Upregulated and downregulated genes were defined using a log2FC threshold of ±0.5. Genes with log2FC > 0.5 were classified as upregulated, and those with log2FC < − 0.5 as downregulated; they are provided between dashed lines; (**B**) Volcano plot of differentially expressed genes in PBMCs before and after ART. Each point represents a gene plotted by log2FC (x-axis) and − log10 adjusted *p*-value (y-axis). Genes significantly downregulated after ART (adj. *p* < 0.05, log2FC < − 0.5) are shown in blue, and upregulated genes (adj. *p* < 0.05, log2FC > 0.5) in red. Gray points are non-significant.

Eighty-seven differentially expressed genes were identified using a paired DESeq2 analysis. Statistical significance was determined based on a Benjamini–Hochberg adjusted *p*-value threshold of < 0.05. Genes with log2FC > 0.5 were classified as upregulated, and those with log2FC < −0.5 as downregulated (Figure 2). Among these, 20 genes were upregulated and 67 were downregulated.

Figure 2A displays the distribution of log2FC among the differentially expressed genes. Upregulated genes exhibited a broader range of log2FC values, while downregulated genes showed a more concentrated distribution, with most genes demonstrating moderate decreases in expression levels.

The volcano plot (Figure 2B) revealed a clear predominance of downregulated genes following therapy, particularly immune-related genes. Upregulated genes were fewer in number and exhibited greater variability in log2FC.

In the following paragraphs, we discuss the genes that are labeled by name in the volcano plot (Figure 2B), including downregulated ISG/immune markers (IFI27, SIGLEC1, CCL2) and the few upregulated coding genes (FCER1A, FCGBP) together with several RPS/RPL-related pseudogenes.

Most of the upregulated genes were annotated as pseudogenes, including those related to ribosomal protein families (RPS and RPL) and pseudogenes derived from elongation factor 1-alpha-1 (EEF1A1). In addition, a few protein-coding genes such as FCER1A (high-affinity IgE receptor subunit alpha) and FCGBP (Fc fragment of IgG binding protein) were also upregulated following ART. In contrast, a marked downregulation was observed in genes involved in antiviral defense mechanisms, including IFI27, SIGLEC1, OTOF, and CCL2.

To explore the possibility of identifying additional differentially expressed genes and assess the influence of threshold stringency, alternative cutoffs were also applied. When the adjusted *p*-value threshold was relaxed to <0.1 while keeping |log2FC| > 0.5, the number of differentially expressed genes increased substantially, yielding 177 genes in total with 41 upregulated and 136 downregulated. Under the relaxed threshold, additional upregulated genes were identified, most of which were annotated as pseudogenes related to ribosomal proteins or EEF1A1, consistent with the trends observed under the more stringent criteria. Notably, newly upregulated protein-coding genes included ARRDC4 (log2FC = 0.67, adj. *p* = 0.05), involved in glucose metabolism and cellular stress responses, and ZNF600 (log_2_FC = 0.56, adj. *p* = 0.085), a zinc finger transcription factor. Among the newly downregulated genes, several were associated with immune regulation and antiviral responses. These included PDCD1 (log_2_FC = −0.66, adj. *p* = 0.078), which encodes an immune checkpoint receptor involved in T cell exhaustion; IRF7 (log_2_FC = −0.66, adj. *p* = 0.099), a key transcriptional regulator of type I interferon responses; and USP18 (log2FC = −1.22, adj. *p* = 0.092), a known negative regulator of interferon-stimulated gene expression. Additional downregulated genes such as CCNA2 (log2FC = −0.93, adj. *p* = 0.065) and CENPE (log2FC = −1.01, adj. *p* = 0.077), both involved in cell cycle regulation and mitotic progression, may reflect reduced immune cell proliferation or activation.

To address potential bias introduced by patients P07 and P08, who exhibited atypical immunological trajectories and transcriptional profiles, we repeated the differential expression analysis after excluding their samples. These two individuals were removed because they showed a decline in CD4^+^ T cell counts following ART, in contrast to the rest of the cohort, and did not follow the expected transcriptional response pattern. Patient P08 also demonstrated a marked decrease in the CD4/CD8 ratio (1.12 to 0.26), driven by CD8^+^ T cell expansion, further suggesting immunological divergence. This sensitivity analysis was performed using the same DESeq2 pipeline with paired comparisons of pre- and post-ART samples from the remaining six participants.

The exclusion of these two patients revealed a more pronounced transcriptional response to ART. Notably, the number of significantly downregulated immunoglobulin genes (based on adj. *p* < 0.01 and |log2FC| > 0.5) increased from 7 to 29, including IGLC7 (log2FC = −2.25), IGHG1 (log2FC = −1.98), IGLV6-57 (log2FC = −1.93), and IGHG3 (log2FC = −1.75), indicating a more consistent suppression of B cell-related transcripts across the remaining participants. In addition, several genes involved in cell cycle regulation and DNA replication, such as CDC20 (log2FC = −1.38), CDCA5 (log2FC = −1.37), CCNA2 (log2FC = −1.15), MCM2 (log2FC = −0.95), and MCM4 (log2FC = −0.91), showed stronger downregulation, suggesting reduced cellular proliferation after ART.

Although some poorly characterized transcripts such as RNU4-2 (log2FC = −3.84) and SMIM43 (log2FC = −2.72) were also among the most downregulated genes, their biological relevance remains unclear.

We also examined selected immune markers of T-cell exhaustion, activation, regulation, proliferation, cytotoxicity, and chemokine signaling. Among activation markers, CD38 showed a significant post-ART decrease (log2FC = −0.99, adj. *p* = 5.14 × 10^−4^). The exhaustion marker PDCD1 (PD-1) also decreased (log2FC = −0.66), reaching trend-level significance (adj. *p* = 0.08). In contrast, class II HLA transcripts (HLA-DRA, -DRB1, -DRB5, -DPA1, -DPB1, -DQA1, -DQB1) exhibited small negative effects (log2FC −0.17 to −0.34) that did not pass the FDR threshold (adj. *p* 0.476 to 1). Regulatory and proliferative readouts were likewise non-significant (FOXP3 log2FC = 0.1, adj. *p* = 1; MKI67 log2FC = −0.67, adj. *p* = 0.552), as were effector and chemokine markers (GZMB log2FC = −0.14, adj. *p* = 1; CCL4 − log2FC = 0.32, adj. *p* = 0.812). Collectively, these results indicate early attenuation of immune activation after ART, with a concurrent trend toward reduced PD-1 signaling, while other regulatory, proliferative, cytotoxic, and chemokine transcripts remain largely unchanged at this time point.

Among activation markers, CD38 showed a significant post-ART decrease (log2FC −0.99, adj. *p* 5.14 × 10^−4^). The exhaustion marker PDCD1 (PD-1) also decreased (log2FC −0.66), reaching trend-level significance (adj. *p* 0.078). In contrast, class II HLA transcripts (HLA-DRA, -DRB1, -DRB5, -DPA1, -DPB1, -DQA1, -DQB1) exhibited small negative effects (log2FC −0.17 to −0.34) that did not meet the FDR threshold (adj. *p* 0.476–1.000). Regulatory and proliferative readouts were likewise non-significant (FOXP3 log2FC +0.10, adj. *p* 1.000; MKI67 −0.67, 0.552), as were effector and chemokine markers (GZMB −0.14, 1.000; CCL4 −0.32, 0.812). Together with the broad downregulation of ISGs, these results indicate early attenuation of immune activation after ART, with a concurrent trend toward reduced PD-1 signaling, while other regulatory, proliferative, cytotoxic, and chemokine transcripts remain largely unchanged at this time point.

### 2.2. Gene Set Enrichment Analysis

**Figure 3:**
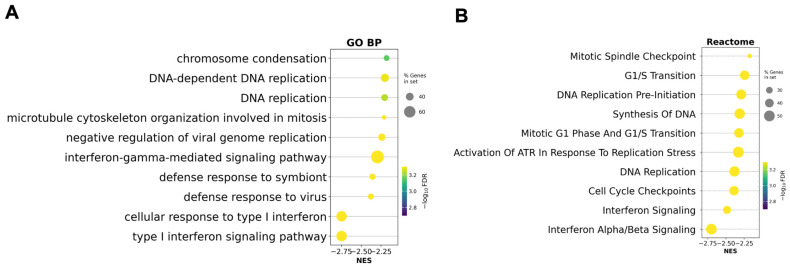
Gene set enrichment analysis (GSEA) based on the preranked gene list after ART. (**A**) Gene Ontology Biological Processes (GO BP) enriched after ART; (**B**) Reactome pathway enrichment analysis enriched after ART. Circle size reflects the proportion of genes in each gene set, and color corresponds to the statistical significance (−log_10_ FDR). Negative normalized enrichment scores (NES) indicate suppression of these pathways in post-ART samples relative to baseline.

To identify pathways downregulated in response to antiretroviral therapy (ART), gene set enrichment analysis (GSEA) was performed using the Gene Ontology (GO) and Reactome pathway databases on the whole expression dataset including all patient samples. The results revealed a significant reduction in the activity of pathways associated with antiviral immune responses and inflammation in post-ART samples (Figure 3). No significantly enriched upregulated pathways were detected, which may be attributed to the limited number and functional ambiguity of the upregulated genes, the majority of which were annotated as pseudogenes.

These findings indicate that ART is associated with the suppression of type I interferon (IFN-I) signaling and related antiviral defense mechanisms. The most significantly downregulated GO terms included ‘cellular response to type I interferon’, ‘type I interferon signaling pathway’, and ‘defense response to virus’. Additional significantly downregulated GO biological processes included pathways involved in cell division and DNA replication, such as ‘chromosome condensation’, ‘DNA-dependent DNA replication’, and ‘microtubule cytoskeleton organization involved in mitosis’. These results suggest that ART may also modulate cell cycle–related processes in PBMCs.

Reactome analysis additionally identified ‘Interferon alpha/beta signaling’ and ‘Interferon Signaling’ as significantly downregulated. These results suggest a shift from a hyperactivated immune state toward a more homeostatic immune profile following viral suppression by ART. Additionally, several cell cycle-related pathways such as ‘Cell Cycle Checkpoint’ and ‘DNA Replication’ were also markedly downregulated, indicating a broader reduction in proliferative activity and DNA replication processes.

To complement the ranked GSEA analysis, functional annotation of the 67 differentially expressed genes was performed using DAVID Bioinformatics Resources 6.8 [19,20]. The enrichment analysis was carried out with Homo sapiens as the background. Consistent with the GSEA results, DAVID identified significant enrichment of pathways involved in antiviral defense and type I interferon signaling, including defense response to virus (False Discovery Rate adjusted *p*-values (FDR) = 6.02 × 10^−24^) and type I interferon signaling pathway (FDR = 1.5 × 10^−8^). Table 1 summarizes the top significantly enriched functional categories, with the full list of annotated terms presented in Supplementary Table S2.

**Figure 4:**
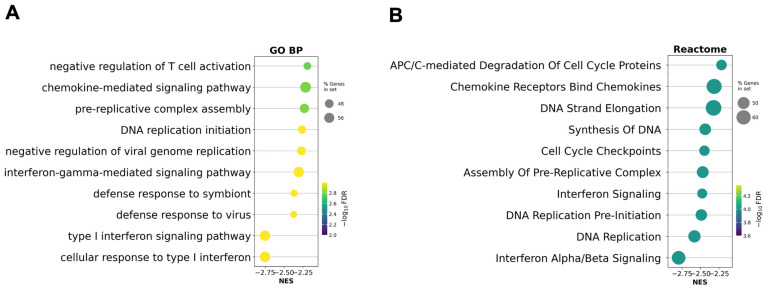
Gene set enrichment analysis (GSEA) based on the preranked gene list after ART excluding outlier patients P07 and P08. (**A**) Gene Ontology Biological Processes (GO BP) enriched after ART; (**B**) Reactome pathway enrichment analysis after ART. Circle size reflects the proportion of genes in each gene set, and color corresponds to the statistical significance (−log_10_ FDR). Negative normalized enrichment scores (NES) indicate suppression of these pathways in post-ART samples relative to baseline.

To assess whether the removal of outlier patients influenced pathway-level interpretation, GSEA was performed on a preranked gene list generated from the updated differential expression analysis after excluding patients P07 and P08 (Figure 4).

Compared to the original analysis, interferon-related and cell cycle–associated pathways remained among the most significantly downregulated, but their relative enrichment scores and ranks changed. GO biological processes such as ‘type I interferon signaling pathway’ and ‘defense response to virus’ remained enriched, while some DNA replication and mitosis-related terms became more prominent. In the Reactome analysis, ‘Interferon Alpha/Beta Signaling’ and ‘DNA Replication’ also remained among the top suppressed pathways, but additional cell cycle checkpoints and APC/C-related modules became more significant.

### 2.3. Results of PPI Network Analysis

To investigate the functional relationships among genes downregulated following ART, we performed an analysis using a protein–protein interaction (PPI) network based on differentially expressed downregulated genes identified between pre- and post-ART samples.

**Figure 5:**
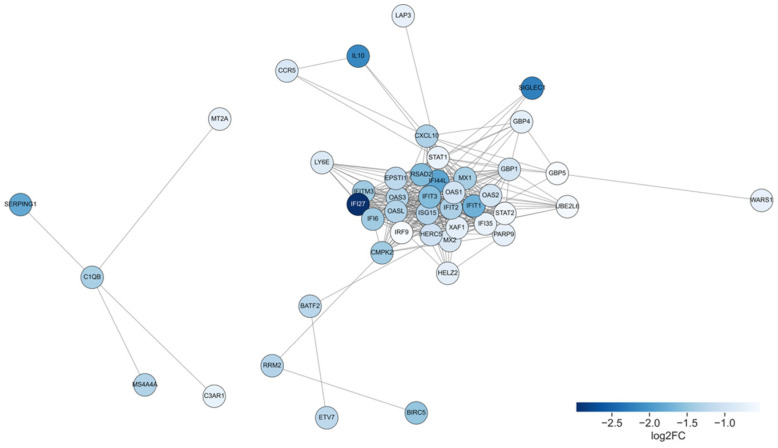
Protein–protein interaction network of downregulated genes after ART. Each node represents a protein encoded by a downregulated gene, and edges denote high-confidence interactions. Node color indicates the magnitude of gene downregulation, represented as log2FC between pre- and post-ART samples: darker blue corresponds to stronger suppression.

The resulting PPI network revealed two major interconnected clusters (Figure 5). The largest cluster predominantly comprised genes involved in type I interferon signaling and innate immune responses. Among the top 20 central nodes within this network, we observed the following nodes corresponding to interferon-stimulated genes (ISGs) ranked by degree centrality (given in brackets): STAT1 (30), IFIT3 (29), ISG15 (28), MX1 (28), IFI44L (27), IFIT1 (26), IFI35 (23), IFI6 (23), MX2 (21), IFI27 (21), IFITM3 (20). Additionally, MX1 is found to be associated with HIV total DNA [21] and MX2 can be involved in the innate immune response to HIV-1 infection [22].

Additional genes such as CXCL10, IL10, and HLA-E were also integrated into the central cluster, highlighting the modulation of cytokine signaling and antigen presentation pathways. A smaller, partially disconnected module included complement components (C1QB, C3AR1, SERPING1).

### 2.4. Clustering Analysis

**Figure 6:**
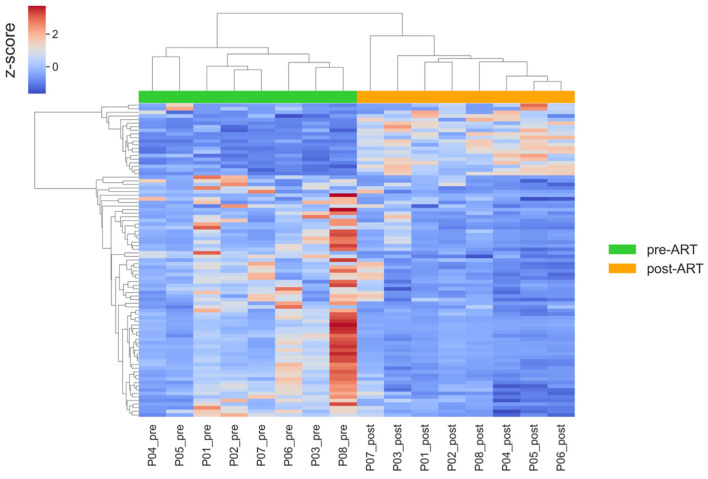
Hierarchical clustering heatmap of differentially expressed genes (|log2FC| > 0.5, adj. *p* < 0.05) before and after ART. Rows represent genes; columns represent patient samples. Values are row-wise z-scores (centered and scaled per gene); the color scale indicates relative expression (higher vs. lower) compared with each gene’s mean.

To characterize the transcriptional shifts associated with ART, we visualized the expression patterns of differentially expressed genes using clustering heatmap (Figure 6).

In general, the overall pattern separates pre- and post-therapy samples. Post-treatment profiles were more homogeneous in comparison with pre-treatment profiles, with reduced expression of immune-related genes across patients. However, samples from P03 and P07 retained relatively higher expression levels of inflammatory and interferon-stimulated genes, which may indicate incomplete suppression of immune activation in these individuals. Patient P08, who had the lowest CD4/CD8 ratio post-ART, exhibited the most pronounced dysregulation in interferon-stimulated gene expression. Similarly, variation in age and baseline immune status may have contributed to the observed heterogeneity. To assess the reliability of our hierarchical clustering, we computed the cophenetic correlation coefficient (CCC) for the dendrograms constructed from differentially expressed genes. The CCC for gene clustering was 0.971, and for sample clustering, it was 0.926. These high values indicate that the dendrograms closely reflect the original pairwise distances among genes and samples, respectively, thereby confirming the robustness and reliability of the observed clustering patterns.

**Figure 7:**
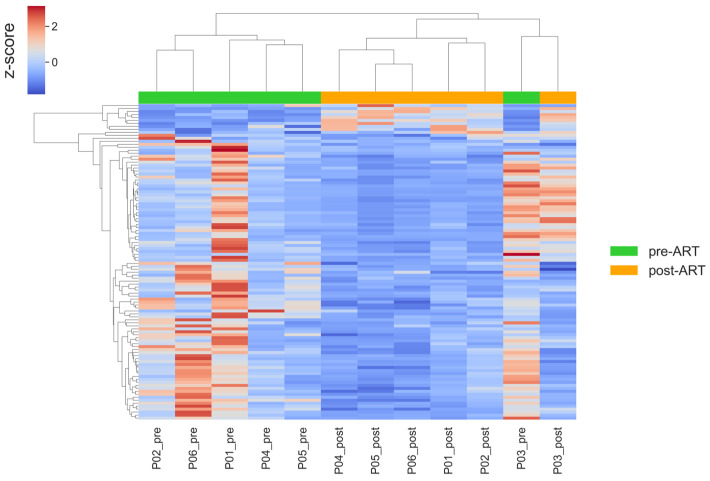
Hierarchical clustering heatmap of differentially expressed genes (|log2FC| > 0.5, adj. *p* < 0.05) before and after ART, after exclusion of samples from patients P07 and P08. Rows represent genes; columns represent patient samples. Values are row-wise z-scores (centered and scaled per gene); the color scale indicates relative expression (higher vs. lower) compared with each gene’s mean.

To visualize the transcriptomic pattern obtained from the differential expression analysis after excluding participants P07 and P08, we constructed a hierarchical clustering heatmap based on the updated DESeq2 results (Figure 7).

The overall clustering structure remained consistent with the main analysis, while the removal of P07 and P08 reduced variability within pre-ART samples, supporting the robustness of the observed transcriptomic trends.

### 2.5. Within-Subject Expression Dynamics of Interferon-Stimulated Genes Before and After ART

**Figure 8:**
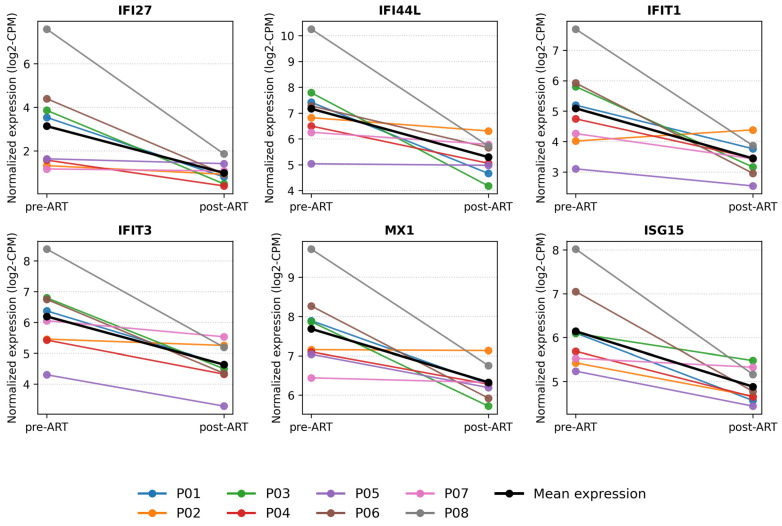
Within-subject expression dynamics of interferon-stimulated genes before and after ART. Paired line plots showing individual (colored lines) and mean (black line) expression changes of representative interferon-stimulated genes (IFI27, IFI44L, IFIT1, IFIT3, MX1, ISG15, IRF7, and PDCD1) before and after antiretroviral therapy.

To better illustrate within-subject transcriptomic changes following ART initiation, we visualized the expression dynamics of representative interferon-stimulated genes using paired line plots (Figure 8). Specifically, we selected six canonical ISGs - IFI27 (log2FC −2.977, adj. *p* 0.00981), IFI44L (−1.854, 0.04827), IFIT1 (−1.726, 0.03545), IFIT3 (−1.601, 1.67 × 10^−5^), MX1 (−1.368, 6.56 × 10^−4^), and ISG15 (−1.279, 6.49 × 10^−4^)—which satisfied adj. *p* < 0.05 and exhibited large, consistent post-ART decreases. Each colored line represents an individual participant, allowing direct comparison of pre- and post-ART profiles, while the black line indicates the cohort mean normalized expression (log2CPM). All selected ISGs demonstrated a consistent decrease in expression after ART, reflecting attenuation of type I interferon signaling and immune activation. These findings highlight the robustness of the paired study design and confirm that ART rapidly normalizes interferon-related transcriptional activity in peripheral blood mononuclear cells.

## 3. Discussion

This study investigated gene expression changes in peripheral blood mononuclear cells following antiretroviral therapy. A total of 67 genes were significantly downregulated, whereas only 20 genes were upregulated in post-ART samples.

ART targets HIV replication by inhibiting key viral enzymes required for completion of the viral life cycle [23]. As a result, ART leads to a reduction in viral load, and consequently, in the levels of viral RNA and DNA that are recognized by innate immune sensors. ART is also associated with reduced activation of immune-related pathways. During the early stages of HIV infection, induction of ISGs and activation of type I interferon (IFN-I) signaling contribute to suppression of viral replication [24,25]. However, chronic ISG activation promotes sustained inflammation [26]. Previous studies have shown that HIV infection induces expression of several ISGs, including RSAD2, ISG15, IFI44L, and IFI27 [27].

Our study focused on the early transcriptomic response in ART-naïve individuals, a clinically relevant population given current treatment guidelines that recommend initiating ART early, regardless of CD4+ count [28,29,30,31]. Importantly, immune dysregulation in HIV infection is not solely reflected by CD4+ T cell depletion. Other immunological markers—such as elevated CD8+ T cell counts and a reduced CD4/CD8 ratio—also reflect immune dysfunction specific to HIV [32,33]. In our cohort, most patients exhibited a decrease in CD8+ T cell counts and an increase in the CD4/CD8 ratio after treatment, indicating improvement in immune homeostasis despite relatively stable CD4+ T cell levels.

In our study, patients following ART demonstrated a significant downregulation of interferon-related responses. The PPI network analysis revealed a large, highly connected module composed of interferon-stimulated genes (ISGs), including STAT1, IFIT3, ISG15, MX1, OAS1, IFI27, IFI44L, and IFI6.

These genes converge within the type I interferon–JAK–STAT signaling cascade. HIV sensing through pattern recognition receptors such as cGAS–STING and RIG-I leads to activation of STAT1 and subsequent ISRE-mediated transcription of ISGs, including ISG15, MX1, IFIT family members (notably IFIT3), and OAS1 [34,35]. In primary myeloid cells, both HIV-1 infection and the viral accessory protein Vpr have been shown to activate this pathway, resulting in increased STAT1 phosphorylation together with coordinated induction of ISG15, MX1, IFIT3/IFI44L, and OAS genes [36]. Similar clustering of ISG15, MX1, IFIT3, OAS1, IFI6, IFI27, and IFI35 within a tightly interconnected type I interferon/RIG-I–like signaling module has also been reported in PBMC transcriptomes in the context of antiviral immune responses, further supporting their functional relatedness [37]. Furthermore, sustained upregulation of this ISG cluster, including ISG15, MX1, OAS1 and STAT1, is a hallmark of untreated HIV-1 infection [38].

Scagnolari et al. (2016) demonstrated that ISG15 expression steadily declined in individuals undergoing long-term antiretroviral treatment, correlating with reductions in markers of immune activation [39]. Sugawara et al. (2021) further demonstrated significantly reduced STAT1 phosphorylation (Y701) in T cells from virally suppressed individuals compared to viremic controls, indicating durable suppression of JAK–STAT signaling after treatment [40].

These findings are also consistent with previous reports by Massanella et al. (2013), who observed a robust downregulation of interferon-inducible genes such as MX1, ISG15, and IFIT3 after 48 weeks of ART, and by Boulware et al. (2010), who similarly demonstrated decreased expression of multiple ISGs—including IFI44L, MX1, IFIT3, IFI27, IFI6, and IFI35—within the first 24 weeks of ART in patients with AIDS [7,8]. Genes within the identified network that have not been previously reported in the context of HIV or ART may represent novel candidates potentially involved in treatment-induced immune modulation and warrant further investigation.

In our PPI analysis, C1QB, SERPING1, and C3AR1 formed a distinct peripheral cluster suggestive of coordinated involvement in complement-mediated immune responses. In HIV infection, the classical complement pathway is activated early via direct recognition of viral envelope proteins such as gp41 by C1q, leading to opsonization, immune cell recruitment, and potential virolysis aimed at limiting viral spread [41,42]. Specifically, C1QB acts as part of the C1q complex initiating the classical complement pathway through direct recognition of viral envelope proteins such as gp41, leading to opsonization and downstream complement activation [43]. SERPING1, encoding the C1 inhibitor, modulates this process by suppressing excessive C1 activation, thereby limiting uncontrolled complement-mediated inflammation; its expression is elevated in viremic individuals and decreases with ART [44]. C3AR1 serves as the receptor for the anaphylatoxin C3a, transducing chemotactic and pro-inflammatory signals that can contribute to HIV-associated immune activation and tissue inflammation [45].

Our transcriptomic findings—marked downregulation of interferon-stimulated genes and pro-inflammatory pathways after 24 weeks of ART—align closely with recent observations in plasma protein biomarkers. Kosmider et al. and De Clercq et al. reported significant reductions, though not full normalization, of CRP, MCP-1, IP-10, TNF-α, and sCD14 within six months of ART [46,47]. Meanwhile, Alagaratnam et al. demonstrated durable decreases in plasma neurofilament light chain (NfL), a marker of neuro-axonal injury, following ART initiated in primary infection—even with treatment interruption—and found that NfL did not rebound despite viral resurgence [48]. In the study by M. Salgado (2024), the author reported proinflammatory markers related to the HIV reservoir elimination [49]. It is important to note that the cited study included patients after allogeneic hematopoietic stem cell transplantation, which may reflect some differences with our study design [49].

Persistent type I interferon (IFN-I) signaling has been implicated in the pathogenesis of chronic HIV infection, where it contributes to sustained immune activation, progressive CD4^+^ T cell loss, and functional exhaustion of T cells [50]. Chronic exposure to IFN-I promotes upregulation of interferon-stimulated genes (ISGs) and drives expression of exhaustion markers such as PD-1 and LAG-3, leading to impaired T cell function and reduced immune competence [50]. In our study, we observed a downregulation of IFN-I–responsive genes following ART. This may reflect a partial restoration of T cell functional capacity and a reduction in immune exhaustion.

However, the downregulation of ISGs may not be exclusively beneficial. In certain contexts, reduced ISG expression could also reflect impaired antiviral surveillance, particularly in light of studies reporting the emergence of IFN-resistant HIV variants during or after ART [51,52]. Although all patients in our cohort achieved sustained viral suppression, this observation warrants further investigation to distinguish between beneficial immune rebalancing and potential loss of innate antiviral responsiveness.

Notably, a recent longitudinal virology study demonstrated that HIV-1 isolates sampled before ART initiation or during chronic infection were relatively sensitive to IFN-I inhibition, whereas viruses emerging during acute infection or following treatment interruption showed the highest IFN-α and IFN-β resistance [51]. This supports our interpretation that ART-mediated suppression of IFN-I pathways may help restore a more controlled and balanced antiviral immune environment.

Some of the unique genes identified in the present study were genes encoding variable regions of immunoglobulin (antibody) heavy (IGHV) and light chains (IGKV/IGLV)—IGHV1-2, IGHV1-46, IGHV4-34, IGKV1-27, IGLV1-47, IGLV3-19, IGLV5-45. HIV-infected individuals may show irregular and dynamically unstable expression patterns of immunoglobulin genes, reflecting substantial disturbances in humoral immune regulation and persistent activation of antibody-mediated responses [53,54]. The observed suppression of immunoglobulin gene expression may reflect a reduction in B-cell activation and antibody production as immune activation subsides post-ART [55,56,57].

In addition, we observed increased expression of pseudogenes corresponding to ribosomal proteins, translation elongation factors, and mitochondrial genes following ART. However, the expression levels of their protein-coding counterparts did not change significantly post-treatment. Retroviral infections, including HIV, have been reported to induce pseudogene transcription [58]. Integration of viral DNA into the host genome can disrupt gene integrity [59], and chronic inflammation, along with activation of DNA repair mechanisms during retroviral infection, may promote genomic instability and pseudogene transcription [60].

The majority of upregulated genes in our transcriptomic profiling belonged to ribosomal protein pseudogenes. Ribosomal pseudogenes, including members of the RPS and RPL families, represent the most abundant pseudogene class in the human genome [61,62]. They are increasingly recognized as potential regulators through competing endogenous RNA (ceRNA) mechanisms and transcript-level modulation, despite lacking protein-coding capacity [63]. Parental RPS and RPL genes encode essential components of the small (40S) and large (60S) ribosomal subunits, respectively, playing a central role in ribosome biogenesis and global protein synthesis by assembling with rRNAs and facilitating translation initiation and elongation [64,65]. Ribosome proteins also participate in innate immune signaling, modulate pathways such as NF-κB and MAPK during infection or stress, and may selectively enhance translation of viral transcripts in host–pathogen interactions [65,66].

We also observed increased expression of two pseudogenes of the elongation factor EEF1A1, namely EEF1A1P17 and EEF1A1P28, following ART. These pseudogene transcripts are proposed to participate in post-transcriptional regulatory mechanisms, such as ceRNA activity, whereby they may sequester shared microRNAs and influence the expression of the parental gene or related pathways [67]. The parental gene EEF1A1 encodes the eukaryotic translation elongation factor 1 alpha 1 (eEF1A1), a core component of the translational machinery responsible for delivering aminoacyl-tRNAs to the ribosome during protein synthesis [68]. Thus, the upregulation of these pseudogenes after ART may reflect transcriptional reprogramming associated with metabolic shifts, potentially enhancing translational capacity or modulating stress-related signaling.

While our dataset revealed only a modest number of upregulated genes, most were ribosomal pseudogenes or poorly annotated loci, which are underrepresented in pathway databases. This likely explains the absence of significantly enriched upregulated pathways in our GSEA results. Therefore, we consider this outcome to reflect both technical limitations in functional annotation and genuine biological dynamics of the early treatment phase.

After ART, patients exhibit signs of immune restoration characterized by increased cellular proliferation and enhanced protein biosynthesis. Massanella M. et al. similarly reported upregulation of certain ribosomal genes, including RPS23, RPL13, and RPL5 [7]. Previous studies comparing untreated HIV-infected individuals to healthy controls reported decreased expression of several ribosomal proteins, such as RPS27, RPL18A, RPL8, RPL26, RPL4, and RPS21, possibly reflecting impaired translational activity during active infection [69]. Furthermore, some studies have described post-ART upregulation of cysteine metabolism pathways, which may support redox balance and enhance T cell function in individuals exhibiting rapid immune recovery [70].

In the study by Massanella M. et al. (2013), the authors reported 4157 differentially expressed genes in PBMCs following 48 weeks of ART, a magnitude much greater than the 87 genes detected in our study after 24 weeks [7]. Several factors may contribute to this discrepancy: a larger cohort size (32 vs. 8), longer ART duration allowing more extensive transcriptomic remodeling and use of microarray technology optimized for detecting known transcripts.

In the study by Massanella M. et al., the upregulation of the JAK-STAT signaling pathway was observed [7]. During acute HIV infection, activation of the JAK/STAT signaling pathway contributes to antiviral immunity via interferon-induced gene expression [71,72]. However, in the chronic phase, sustained activation of this pathway has been associated with persistent inflammation, immune activation, and T-cell dysfunction [72]. The activation of the JAK-STAT signaling pathway in individuals following ART can also be associated with immune reconstitution [71].

However, in our cohort, no significant activation of these pathways was observed. This may reflect the relatively early phase of ART response in our cohort, as a 24-week duration of ART may be insufficient to elicit the metabolic changes observed during more advanced phases of immune recovery. Immune reconstitution following ART is a gradual process that typically occurs in two phases: an initial rapid increase in CD4+ T-cell counts during the first 3–6 months, followed by a slower, prolonged recovery phase that may continue for several years [73,74]. The extent and timing of immune restoration can vary considerably between individuals, depending on baseline immune status and other host factors [73].

The absence of a clear separation between pre- and post-ART samples in the PCA plot likely reflects the relatively early stage of treatment captured in our cohort. At 24 weeks after ART initiation, the dominant transcriptomic changes are primarily characterized by suppression of interferon-stimulated genes and inflammatory signaling pathways. These immunosuppressive shifts, although biologically meaningful, may not produce extensive global variance detectable by unsupervised dimensionality reduction techniques such as PCA. Moreover, participants initiated ART with relatively high CD4^+^ T cell counts and without advanced clinical manifestations of immune dysregulation, further limiting the extent of global transcriptional divergence. Under such conditions, inter-individual heterogeneity can confound treatment-related effects in PCA, resulting in overlapping sample distributions despite coherent paired differential expression patterns.

This interpretation is also supported by findings from Zhou et al. (2023), who investigated PBMC transcriptomes in patients with HIV receiving ART for 3–6 months [12]. Similar to our findings, they observed partial normalization of both coding and non-coding RNA expression. However, inflammatory and structural pathways remained incompletely resolved, indicating that longer treatment durations may be necessary to achieve full molecular recovery.

Our study identified 87 differentially expressed genes following 24 weeks of ART, a number considerably lower than the 1412 upregulated genes and 521 downregulated differentially expressed genes reported by Zhou et al. after 3–6 months of treatment [12]. This discrepancy may be a result of methodological differences, including the use of different statistical thresholds for defining significance. Moreover, Zhou et al. [12] included both protein-coding and long non-coding RNAs (lncRNAs) in their analysis, whereas our workflow focused only on well-characterized genes with standardized gene names, excluding most lncRNAs. Our analysis prioritized biological interpretability by focusing on well-annotated, protein-coding genes with established functional relevance.

Our analysis revealed a predominance of downregulated genes after 24 weeks of ART, in line with earlier findings by Boulware et al. [8]. In that study, gene expression profiling at 4, 8, and 24 weeks of ART revealed a higher number of downregulated than upregulated genes, and the majority of transcripts were either not expressed or unaffected by treatment [8]. This timing aligns with clinical guidelines, which note that substantial immunologic recovery, including increases in CD4^+^ T cell counts, is typically observed within the first 24 weeks of effective ART, although full immune restoration may continue for years thereafter [75].

In contrast, longer-term studies such as Massanella et al. (2013), which analyzed samples after 48 weeks of ART, have reported broader transcriptomic shifts, including upregulation of metabolic and biosynthetic pathways, reflecting more advanced immune reconstitution [7]. Therefore, our 24-week time point likely captures an “early” signature characterized by the attenuation of inflammatory and interferon-stimulated pathways, preceding the activation of restorative transcriptional programs seen at later stages.

Second, the homogeneity of our cohort—free from co-infections or comorbidities—may have contributed to the obtained results. Finally, our conservative filtering criteria and focus on annotated genes may have limited the detection of moderate upregulation signals. Together, these factors may explain the asymmetry in gene expression changes in our dataset.

Comparison of transcriptomic profiles revealed individual expression profiles corresponding to the peculiarities of response to therapy; in particular, patients P07 and P08 showed a decline in CD4+ T cell counts from 675 to 575 cells/μL and from 642 to 516 cells/μL, respectively, over the 24-week period, indicating a suboptimal immunological response to ART [46]. Patient P08 exhibited an atypical pre-therapy expression profile of immune-related genes, indicative of underlying immune dysfunction, and experienced a pronounced decline in the CD4/CD8 ratio (from 1.12 at baseline to 0.26 post-ART) driven by a disproportionate expansion of CD8^+^ T cells (from 576 to 1990 cells/μL). Despite virological suppression, we can only suppose that this pattern represents an atypical immune response, possibly linked to persistent or bystander activation [76,77]. This observation may also point to underlying host-specific, genetic, or environmental factors influencing immune recovery, which remain to be elucidated.

Patient P07, by contrast, showed reduced CD4^+^ counts with minimal improvement in the CD4/CD8 ratio and elevated interferon-stimulated/inflammatory transcripts after ART, features consistent with an immunologic non-response associated with incomplete suppression of immune activation [17]. Notably, these two individuals did not differ markedly from the rest of the cohort in terms of age, estimated duration of infection (≤50 days), or other clinical parameters, suggesting that their atypical responses are unlikely to be explained by baseline characteristics alone.

One of the main limitations of our study is the small cohort size, which reduces statistical power and limits the generalizability of our findings. An additional limitation of this study is the absence of experimental validation of the transcriptomic findings, such as quantitative PCR analysis of selected genes. To overcome this limitation, we performed an extensive literature-based validation of our results. Nevertheless, the use of a paired-sample design and rigorous statistical thresholds provides confidence in the reported differential gene expression patterns.

## 4. Materials and Methods

### 4.1. Clinical Characteristics

RNA sequencing data were obtained from peripheral blood samples of patients with HIV participating in a study organized by the Institute of Biomedical Chemistry, Central Research Institute of Epidemiology and the Krasnodar Clinical Center for HIV Prevention and Treatment. The study included eight male participants aged between 18 and 35 years who had not previously received antiretroviral therapy. None of these participants had comorbidities or secondary infections that might confound the immune response or gene expression profiles. Clinical parameters of patients, including age, CD4+ and CD8+ T-cell counts, plasma viral load, are shown in Table 2. Participants were administered combination ART, which included the nucleoside reverse transcriptase inhibitors tenofovir disoproxil fumarate (TDF) and lamivudine (3TC) and the integrase inhibitor dolutegravir (DTG). PBMCs were collected at baseline and after 24 weeks of ART. The study was approved by the local ethics committee, and written informed consent was obtained from all participants prior to enrollment.

### 4.2. RNA-Seq Data Generation and Preprocessing

The post-therapy samples were collected after 24 weeks of treatment, while the pre-therapy samples were collected before its initiation. Total RNA was extracted from peripheral blood mononuclear cells (PBMCs) and used to construct poly(A)+ RNA libraries. The isolation of live cells from dead cells was performed as a part of the standard sample preparation protocol; however, the resulting sample may contain a small number of dead cells, which could potentially influence gene expression patterns. The quality and quantity of the isolated total RNA were checked using a BioAnalyser and RNA 6000 NanoKit (Agilent, Santa Clara, CA, USA). Quality of total RNA fractions was assessed using an Agilent Tapestation to calculate an RNA Integrity Number (RIN). Higher RIN values indicate better integrity of total RNA, with the highest RIN value being 10. Only RNA samples with a RIN >8 were used for sequencing analysis. Library quality was assessed using the Agilent High Sensitivity DNA Kit (Agilent Technologies). Sequencing was performed on the Illumina HiSeq1500 platform, generating a minimum of 10 million single-end reads of 50 nucleotides per sample, which corresponds to a standard depth considered sufficient for RNA sequencing according to the literature [78,79]. The quality of the obtained FASTQ files was assessed by FastQC software (version 0.10.1). The quality was high for all samples, making them suitable for further analysis. On average, a median Phred score was higher than 30 for all bases in the read, and most of the reads had mean Phred scores more than 36. The observed GC distribution was similar to the theoretical distribution, indicating that the samples were contaminant-free.

Raw sequencing reads were aligned to the human reference genome (GRCh38) using the STAR aligner (version 2.7.9a). Gene annotation was based on Ensembl release 99. Gene-level read counts were obtained by mapping aligned reads to annotated gene features using STAR’s built-in quantification mode. About 98.5% of the reads were mapped to genes, and 84.5% of them were mapped to unique genes.

### 4.3. Differential Gene Expression Analysis

Differential expression analysis was performed using the DESeq2 package (v1.28.1) in R with a paired design (~patient + condition), allowing comparison of pre- and post-ART samples within each individual. The pre-ART samples were used as the reference group for statistical modeling. Genes with an adjusted *p*-value (Benjamini–Hochberg correction) < 0.05 and absolute log2FoldChange (log2FC) > 0.5 were considered differentially expressed.

To investigate transcriptomic variation between pre- and post-ART samples, principal component analysis (PCA) was performed using the scikit-learn library (version 1.6.1) in Python. Raw counts were converted to counts per million (CPM), and low-abundance features were removed (CPM ≥ 1 in ≥3 samples). The data were log2-transformed [log2(CPM + 1)], and the 250 most variable genes were selected based on variance across samples. Prior to PCA, gene-wise z-scoring was applied.

Gene set enrichment analysis (GSEA) was performed using the GSEApy library (version 1.1.5) in preranked mode using the complete set of genes [80]. All genes included in the DESeq2 results were ranked by log2FC values [80]. Gene Ontology (GO) and Reactome pathways were used for analysis [81,82].

Differentially expressed genes were grouped into clusters using hierarchical clustering with the average linkage method and Spearman distance as the dissimilarity metric. To remove the influence of differences in expression level between genes, the data were normalized across rows (genes) using z-normalization. Data analysis and visualization were performed using Python (version 3.11.0) and the libraries pandas (version 2.2.3), seaborn (version 0.12.2), matplotlib (version 1.24.4), and methods from the scipy library (version 1.10.1).

### 4.4. PPI Network Analysis

We performed a functional analysis for 67 genes with decreased expression after antiretroviral therapy (ART) based on the protein–protein interaction (PPI) using the STRING database [83]. To ensure reliability of detected interactions, a minimum confidence score of 0.7 was applied, corresponding to the “high confidence” threshold recommended by STRING documentation and was empirically chosen to balance network interpretability and stringency [83]. Confidence scores in STRING represent the estimated probability of a true protein–protein interaction, calculated by integrating multiple evidence types and benchmarking using KEGG pathway co-membership [83]. The PPI network was visualized and analyzed using the NetworkX Python library.

To identify central (hub) genes within the network, we calculated the degree centrality of each node using NetworkX’s degree function, which measures the number of direct connections (edges) associated with a given gene. Genes with the highest degree were considered central to the network structure and potentially more influential in mediating ART-induced immunological shifts. Gene nodes were colored based on log2FC values to represent expression differences between pre- and post-ART states.

## 5. Conclusions

Our transcriptomic analysis of paired PBMC samples from HIV-infected individuals revealed that antiretroviral therapy leads to broad suppression of interferon-stimulated gene expression and antiviral pathways, consistent with reduced immune activation following viral suppression. In addition to well-characterized immune modulatory effects, we observed an unexpected upregulation of several pseudogenes related to protein biosynthesis, suggesting a potential transcriptional shift toward cellular recovery and metabolic adaptation.

As a result of the analysis, we observed the significant downregulation of interferon-stimulated genes (ISGs) and the suppression of related pathways, including type I interferon signaling, antiviral defense, and innate immune responses. The coordinated downregulation of a tightly interconnected network of ISGs, including such key hubs as STAT1, ISG15, and IFIT3, underscores the central role of the IFN-I/JAK-STAT axis in HIV-driven immune activation and its reversal upon treatment.

Furthermore, we observed a concurrent downregulation of cell cycle and DNA replication pathways, indicating reduced immune cell proliferation following ART. The sensitivity analysis, which excluded immunologically discordant patients, reinforced these findings and revealed a more pronounced suppression of B-cell-related immunoglobulin transcripts, suggesting a global attenuation of both innate and adaptive immune activation. Additionally, we could observe the upregulation of numerous pseudogenes, particularly those derived from ribosomal proteins (RPS, RPL) and the elongation factor EEF1A1. While their functional role remains to be fully elucidated, their increased expression may reflect a transcriptional reprogramming linked to cellular recovery, metabolic adaptation, and potentially the re-establishment of translational machinery as immune activation subsides.

Clinically, these transcriptomic changes correlated with overall virological suppression and, in most patients, improvements in immunological parameters such as the CD4/CD8 ratio. The heterogeneity observed in patients with suboptimal immunological recovery (P07, P08) highlights how transcriptomic profiling can identify individuals with persistent immune dysregulation despite virological control. The unexpected upregulation of pseudogenes associated with protein synthesis opens new avenues for investigating the mechanisms of post-therapeutic cellular recovery. While the small cohort size necessitates validation in larger studies, our findings enhance the understanding of the molecular underpinnings of immune reconstitution and may contribute to developing targeted strategies to optimize long-term health outcomes for people living with HIV.

## Figures and Tables

**Table 1:** Functional annotation of differentially expressed genes using DAVID. The table lists the top enriched biological processes and pathways identified among the downregulated genes after 24 weeks of ART. Count indicates the number of genes associated with a given term. % of gene list refers to the proportion of the input gene list annotated with the term. *p*-value is the modified Fisher Exact *p*-value from DAVID, reflecting the significance of term enrichment. Fold Enrichment represents the ratio of observed to expected gene counts for each term. FDR corresponds to the Benjamini–Hochberg false discovery rate used to correct for multiple testing.

Term	Count	% of Genes	*p*-Value	Fold Enrichment	FDR
Antiviral defense	22	32.87	2.85 × 10^−27^	33.64	9.99 × 10^−26^
Defense response to virus	23	34.33	1.35 × 10^−26^	29.05	6.02 × 10^−24^
Immunity	31	46.27	9.08 × 10^−19^	6.63	1.59 × 10^−17^
Innate immunity	23	34.33	5.81 × 10^−18^	11.18	6.78 × 10^−17^
Negative regulation of viral genome replication	10	14.92	1.38 × 10^−14^	69.17	3.08 × 10^−12^
Response to virus	12	17.91	6.59 × 10^−14^	32.61	9.80 × 10^−12^
Innate immune response	19	28.36	1.45 × 10^−13^	10.15	1.61 × 10^−11^
Interleukin-27-mediated signaling pathway	6	8.96	1.67 × 10^−11^	228.26	1.49 × 10^−9^
Antiviral innate immune response	9	13.43	3.30 × 10^−11^	42.14	2.45 × 10^−9^
Type I interferon-mediated signaling pathway	8	11.94	2.35 × 10^−10^	48.70	1.50 × 10^−8^

**Table 2:** Clinical characteristics of patients’ baseline.

Label	Age (Years)	CD4+ T Lymphocyte (Count/µL) Before/After ART	CD8+ T Lymphocyte (Count/µL) Before/After ART	CD4/CD8 Ratio Before/After ART	Plasma Viral Load (Copies/mL) Before/After ART
P01	28	782/1070	1024/902	0.76/1.19	154,272/<20
P02	34	540/802	1062/1055	0.51/0.76	64,368/<20
P03	34	413/913	822/1161	0.50/0.79	27,942/<20
P04	33	673/837	898/963	0.75/0.87	3825/<50
P05	18	705/779	914/884	0.77/0.88	262,686/<50
P06	35	543/582	1195/864	0.45/0.67	8821/<50
P07	24	675/575	884/698	0.76/0.82	31,635/<50
P08	26	642/516	576/1990	1.12/0.26	81,743/<50

## Data Availability

The RNA-seq read count data and differential expression statistics generated in this study are provided in the Supplementary Materials (Supplementary Table S1).

## Supplementary Materials

The following supporting information can be downloaded at https://www.mdpi.com/article/10.3390/ijms262110678/s1.

## Abbreviations

The following abbreviations are used in this manuscript:

HIV	Human Immunodeficiency Virus
ART	Antiretroviral Therapy
PBMCs	Peripheral Blood Mononuclear Cells
TDF	Tenofovir Disoproxil Fumarate
3TC	Lamivudine
DTG	Dolutegravir
RNA-seq	RNA sequencing
DEGs	Differentially Expressed Genes
GSEA	Gene Set Enrichment Analysis
PCA	Principal Component Analysis
PPI	Protein–Protein Interaction
STRING	Search Tool for the Retrieval of Interacting Genes/Proteins
GO	Gene Ontology
NES	Normalized Enrichment Score
FDR	False Discovery Rate
DAVID	Database for Annotation, Visualization and Integrated Discovery
ISGs	Interferon-Stimulated Genes
IFN-I	Type I Interferon
JAK–STAT	Janus Kinase–Signal Transducer and Activator of Transcription
