# Supplementary figures and images for: Early Transcriptomic Signatures of Immune Response Modulation Following Antiretroviral Therapy in HIV-Infected Patients
Source: Int J Mol Sci. 2025 Nov 2;26(21):10678. doi: 10.3390/ijms262110678 (PMC12608518; doi:10.3390/ijms262110678)

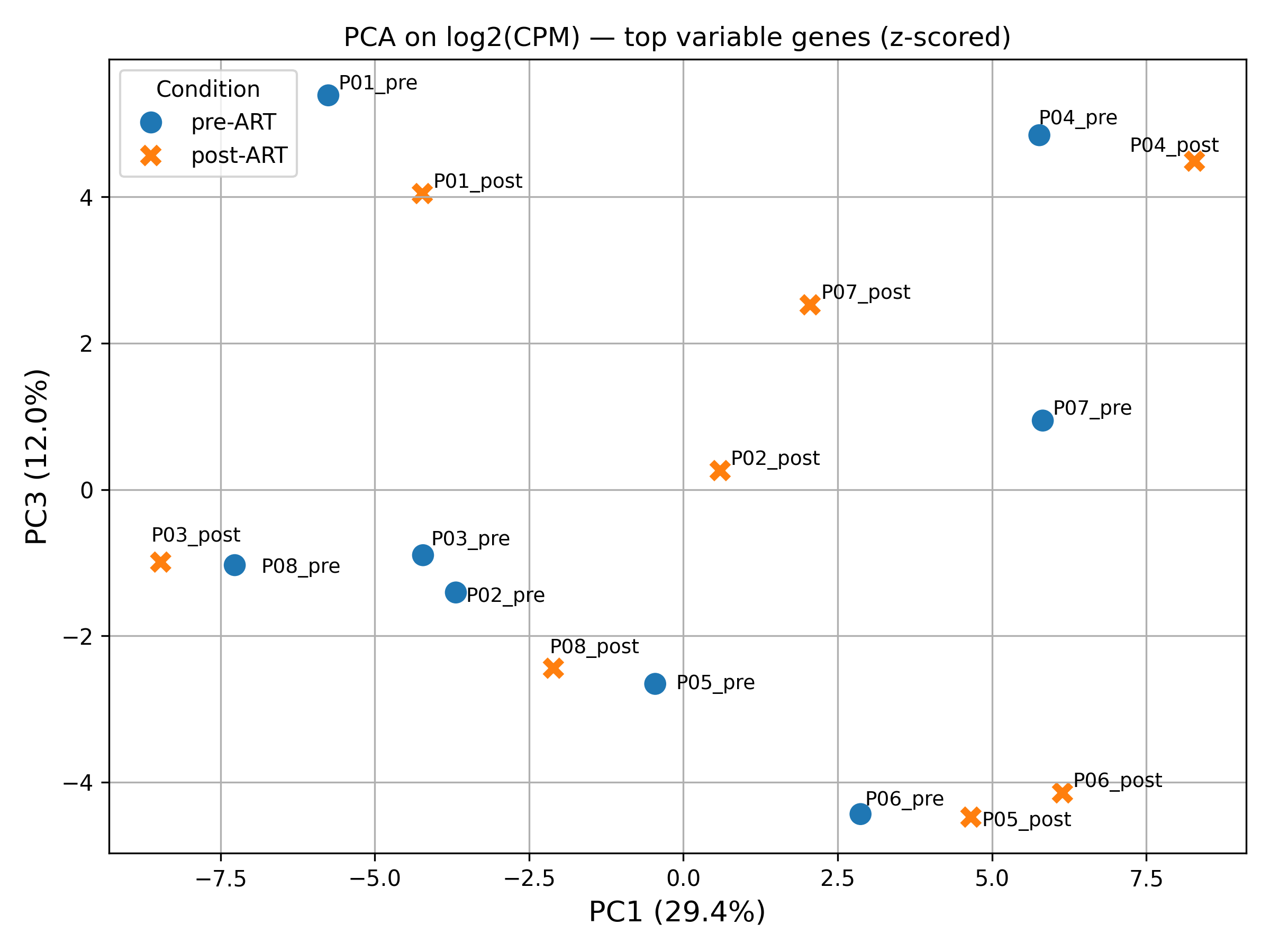

Supplement: Supplementary file 1 [file ijms-26-10678-s001.zip › ijms-3931030-supplementary/Supplementary Materials/Figure S1.tiff]
